# circFOXK2 Stabilizes STMN1 mRNA via PABPC1 to Promote the Progression of NSCLC

**DOI:** 10.1002/cam4.70729

**Published:** 2025-02-27

**Authors:** Wei Chen, Weijun Zhao, Xianqiao Wu, Tianzheng Fang, Ziyuan Chen, Zixuan Chen, Wenmin Su, Xiaodong Zhao, Yuanyuan Hu, Yiping Xu, Shuai Fang, Chengwei Zhou

**Affiliations:** ^1^ Thoracic Surgery Department The First Affiliated Hospital of Ningbo University Ningbo Zhejiang People's Republic of China; ^2^ Ningbo University School of Medicine Ningbo Zhejiang People's Republic of China

**Keywords:** circFOXK2, NSCLC, PABPC1, RNA–protein interaction, STMN1

## Abstract

**Background:**

Lung cancer has a notably high incidence and mortality rate, and understanding its occurrence and development is crucial for effective treatment. Circular RNA is closely associated with tumor progression, playing a role in tumorigenesis and development by regulating gene expression and influencing cell proliferation and apoptosis. This study aims to explore the circFOXK2 role in NSCLC occurrence and development and to elucidate its underlying mechanisms.

**Methods:**

qRT‐PCR and Western Blot analyzed the expressions of circFOXK2, STMN1, and PABPCA in NSCLC cell lines, as well as their relationships. The roles of circFOXK2 and STMN1 in the proliferation, invasion, and migration of NSCLC cells were assessed through CCK8, EDU, and Transwell experiments. RNA pulldown assays with mass spectrometry elucidated the RNA‐binding proteins of circFOXK2. Subcutaneous tumorigenesis and tail vein lung metastasis experiments analyzed the impact of circFOXK2 on tumor growth and metastasis in vivo.

**Results:**

In this study, we identified circFOXK2, which is significantly overexpressed in NSCLC, through bioinformatics screening. Elevated levels of circFOXK2 enhance the growth and metastasis of NSCLC cells. Furthermore, through experiments such as co‐IP and mass spectrometry, we found that circFOXK2 interacts with PABPC1 to form a complex, which correlates positively with the progression and metastasis of tumors. Simultaneously, the circFOXK2/PABPC1 complex increases the stability of STMN1 mRNA, thereby promoting the transcription and translation of STMN1. Our experimental results indicate that the oncogenic effect of circFOXK2 requires the assistance of STMN1.

**Conclusions:**

In summary, we have demonstrated the significant role of circFOXK2/PABPC1 in regulating STMN1 expression in NSCLC.

## 1 Introduction

Lung cancer (LC) is the most common and deadliest tumor worldwide [1, 2, 3]. The two main types of lung cancer are non‐small cell lung cancer (NSCLC) and small cell lung cancer (SCLC) [4]. Among them, NSCLC constitutes the majority of cases [5]. Despite some success with targeted therapies and immunotherapy [6, 7], the 5‐year survival rate for NSCLC patients continues to be significantly low [8, 9]. Consequently, conducting in‐depth research into NSCLC occurrence and development mechanisms is essential to improve clinical treatments.

Circular RNA (circRNA) is a noncoding RNA class widely present in cells [10]. CircRNA has unique expression patterns, molecular stability, and specificity, and is commonly distributed in the human body [11, 12, 13]. Recent research indicates that circRNA can function as a biomarker for cancer detection and diagnosis [14, 15]. The dysregulation of circRNA can lead to tumorigenesis and migration of various cancers, making it an effective target for cancer treatment [16, 17].

The occurrence and progression of cancer have been linked to the dysregulation of various genes [18, 19]. Stathmin (STMN1) is overexpressed in various cancers, contributing to tumor growth, metastasis, and poor prognosis [20, 21]. Given its function as an oncogene, STMN1 is often referred to as an oncoprotein [22]. These discoveries underscore the critical role of STMN1 in the onset and progression of tumors [23, 24]. However, the molecular mechanisms by which STMN1 regulates non‐small cell lung cancer (NSCLC) remain unclear.

The Poly(A)‐binding protein (PABP) family is typically seen as a protective barrier for the mRNA poly(A) tail, influencing various aspects of mRNA translation and stability [25]. PABP cytoplasmic 1 (PABPC1) is extensively present in the cytoplasm of eukaryotic cells, mediating mRNA translation, degradation, and cytoplasmic polyadenylation, making it play an important role in various cellular activities [26, 27]. Increasingly, research has shown that PABPC1 is aberrantly expressed in numerous tumors and contributes to carcinogenesis [28, 29]. Yet, the role of PABPC1 remains largely unexplored in NSCLC.

For this research, we pinpointed circRNAs originating from FOXK2, specifically hsa_cir c_0046430, dubbed circFOXK2, by examining circRNA expression patterns in NSCLC. circFOXK2 was highly upregulated in NSCLC compared to control non‐tumor tissues, and the silencing of STMN1 contributed to the downregulation of circFOXK2. Functionally, circFOXK2 influences NSCLC cells by binding directly to PABPC1 and forming circRNA‐protein complexes. Our findings indicate that circFOXK2 may play a role in NSCLC progression, potentially acting as a tumor suppressor.

## 2 Materials and Methods

### 2.1 Clinical Specimens and Cell Culture

Tissue samples from lung cancer patients, consisting of both tumor and adjacent normal tissues, were sourced from the First Affiliated Hospital of Ningbo University. The selected samples were gathered from patients who had not undergone radiotherapy, chemotherapy, or targeted therapy and were collected through surgical resection. Participants were granted written informed consent, and the research protocol received approval from the Medical Ethics Committee of the First Affiliated Hospital of Ningbo University.

HBE, A549, NCI‐H1299, NCI‐H1650, and SPC‐A1 cell lines were acquired from the Chinese Academy of Sciences (Shanghai, China). The cells were cultured in RPMI‐1640 medium supplemented with 10% fetal bovine serum (Gibco, USA) and 1% penicillin/streptomycin (Solarbio, China). Cultivation was conducted in a humidified incubator at 37°C and 5% CO2.

### 2.2 Cell Transfection

Specific overexpression plasmids and lentiviruses for circFOXK2, STMN1, and PABPC1, along with their corresponding control vectors, were purchased from Tsingke (China, Beijing). In addition, siRNAs for circFOXK2, STMN1, and PABPC1, along with the corresponding control siRNAs, were also acquired from Tsingke (China, Beijing). Cells were transfected using Lipofectamine 2000 (Invitrogen, USA) following the manufacturer's protocol.

### 2.3 RNA Isolation, qRT‐PCR, RNase R Treatment, and Sanger Sequencing

RNA extraction from tissues and cells was performed utilizing Trizol (Ambion, USA). Subsequently, reverse transcription into cDNA was accomplished using NovoScript Plus All‐in‐one 1st Strand cDNA Synthesis SuperMix (gDNA Purge) (Novoprotein, China). qRT‐PCR analysis was then carried out utilizing the NovoStart SYBR qPCR SuperMix Plus (Novoprotein, China). For RNase R treatment (Epicenter Technologies, USA), 2 μg of RNA was incubated with or without 5 U of RNase R at 37°C for 30 min, and qRT‐PCR then measured the expression of circFOXK2 and its parent gene FOXK2. The amplified circFOXK2 PCR products were separated on a 1% agarose gel, and the back‐splice junction of circFOXK2 was confirmed by Sanger sequencing. The primer details can be found in Table S1.

### 2.4 Actinomycin D Treatment

A549 and NCI‐H1299 cells were plated in 12‐well plates, and after 24 h, 5 μg/mL actinomycin D (Sigma, USA) was administered to the wells. Total RNA was extracted at different time points post‐treatment, and qRT‐PCR was used to measure the expression levels of circFOXK2 and FOXK2.

### 2.5 Cell Viability Assay

Cell viability was measured using the CCK8 reagent (NCM, China). The transfected A549 and NCI‐H1299 cells were plated in a 96‐well plate at 3 × 10^3^ cells per well with three replicates per group. At 0, 24, 48, 72, and 96 h after culture, each well received 10 μL of CCK‐8 solution. Following a 3‐h incubation in an incubator, the absorbance at 450 nm was measured using a microplate reader.

### 2.6 EDU Assay

As per the manual, cell proliferation was assessed using the BeyoClick EdU‐555 Cell Proliferation Kit (Beyotime, China). The transfected A549 and NCI‐H1299 cells from each group were plated in a 24‐well plate. After 24 h of culture, EDU reagent was added, and following an additional 4 h of incubation, the cells were stained with Alexa Fluor 555 dye solution and Hoechst 3342. An inverted fluorescence microscope was used to assess the percentage of EDU‐positive cells.

### 2.7 Migration and Invasion Assays

For the migration assay, in the upper chamber, 200 μL of serum‐free medium containing 4 × 10^4^ transfected A549 and NCI‐H1299 cells was added, and 750 μL of medium with 10% FBS was added to the lower chamber. Following a 48‐h incubation, cells on the lower surface of the upper chamber were fixed using 4% paraformaldehyde and subsequently stained with 1% crystal violet. Photographs were taken with a microscope, and the cells were counted. The invasion assay followed the same protocol but with matrigel (Thermo, USA) spread in the upper chamber prior to cell addition.

### 2.8 Western Blotting

Cells from each group were broken down using RIPA lysis buffer with 1% PMSF (Solarbio, China), and total protein was obtained from the supernatant after centrifugation. Proteins were separated using 10% SDS‐PAGE and then transferred to PVDF membranes. The membranes were then incubated with primary antibodies overnight at 4°C, followed by a 2‐h incubation with secondary antibodies. Proteins were visualized using an ECL kit (advansta, China). GAPDH (Abcam, USA) was used as an internal control. Primary antibodies for STMN1 (Abcam, England), E‐cadherin (Proteintech, China), N‐cadherin (Proteintech, China), Vimentin (Proteintech, China), and PABPC1 (Abcam, USA) were used.

### 2.9 Immunohistochemistry

Firstly, tumors were paraffin‐embedded, dehydrated, and cut into sections before being mounted onto slides. Paraffin was removed using xylene, and the sections were treated with a series of ethanol solutions in decreasing concentrations. Then, the slides were incubated with 3% hydrogen peroxide for 15 min, followed by a 30‐min blocking with a blocking solution. Primary antibodies for Ki67, E‐Cadherin, and STMN1 were incubated with the sections overnight at 4°C. Subsequently, there was a subsequent incubation with secondary antibodies at room temperature. In the end, images were captured under a microscope after staining the sections with DAB solution and hematoxylin.

### 2.10 RNA Pulldown Assay

First, biotin‐labeled circFOXK2 sense or antisense probes were engaged in a 3‐h incubation with magnetic beads to prepare probe‐coated magnetic beads. Subsequently, A549 and NCI‐H1299 cells were collected and lysed using ultrasound, followed by an overnight incubation with the probe‐coated magnetic beads at 4°C. The magnetic beads were washed, and the target proteins were purified and eluted. RNA–protein interactions were assessed through experiments such as Western blot, silver staining, and mass spectrometry techniques.

### 2.11 RIP Assay

The interaction between PABPC1 and circFOXK2 or STMN1 was analyzed using the Megna RIP RNA‐Binding Protein Immunoprecipitation Kit (Millipore, USA). A549 and NCI‐H1299 cells were collected and cross‐linked with 254 nm ultraviolet light, followed by lysis in RIP lysis buffer. The lysates were then co‐precipitated with magnetic beads preincubated with PABPC1 (Abcam, USA) or IgG (Abcam, USA) antibodies. Co‐precipitated RNAs were quantitatively assessed using qRT‐PCR.

### 2.12 RNA Fluorescence In Situ Hybridization (FISH)

A549 and NCI‐H1299 cells fixed on coverslips with 4% paraformaldehyde were treated with 0.5% Triton X‐100 at 4°C for 10 min, followed by hybridization with a hybridization mixture containing a Cy3‐labeled circFOXK2 probe and incubation overnight at 37°C. The slides were subsequently rinsed with a buffer solution to remove unbound probes, then stained the cells with DAPI. Fluorescent images were obtained using a laser‐scanning confocal microscope.

### 2.13 Nuclear‐Cytoplasmic Separation

Cellular and nuclear RNA from A549 and NCI‐H1299 cells was separated using the instructions of the Cell and Nuclear RNA Purification Kit (Norgenbiotek, Canada). qRT‐PCR was employed to assess the relative expression levels of circFOXK2, with U6 and GAPDH as internal controls for the nucleus and cytoplasm, respectively.

### 2.14 RNA Sequencing

Three pairs of lung cancer samples, comprising tumor tissues and corresponding adjacent tissues, were selected to extract total RNA using Trizol and identify differentially expressed circRNAs. Differential expression of circFOXK2 was selected for subsequent research. Total RNA from A549 and NCI‐H1299 cells, with circFOXK2 silenced and controls, was extracted using Trizol and further processed by rRNA removal and RNase R digestion, then reverse transcribed into cDNA. After cDNA purification, sequencing was performed (OE Biotech, China).

### 2.15 Animal Experiments

Animal experiments were conducted using four‐week‐old BALB/c nude mice obtained from a supplier. To study tumor formation in vivo, mice were randomly assigned to either the sh‐NC or sh‐circFOXK2 groups, each consisting of 6 mice. A549 cells were subcutaneously injected into the mouse abdomen (1 × 10^7^, 100 μL). Four weeks after inoculation, the mice were euthanized, and the tumor volume was measured. Tumor volume was determined using the formula: length × width^2^ × 0.52.

For the tumor metastasis model, A549 cells stably transfected with sh‐circFOXK2 and sh‐NC were injected into the tail vein of nude mice (5 × 10^6^, 100 μL). Five weeks later, the mice were euthanized, and their lungs were harvested.

### 2.16 Statistical Analysis

Statistical analysis was conducted with GraphPad Prism 9.1 (GraphPad Software). Mean ± standard deviation (SD) was used to represent the results, with differences between two unrelated groups evaluated using a two‐tailed Student t‐test and matched group data by paired t‐test. The area under the curve (AUC) from ROC curve analysis was used to evaluate its capability to differentiate between tumor and normal tissue. Pearson correlation coefficient was utilized to evaluate the relationship between STMN1 and circFOXK2 expression levels. A *p* value < 0.05 was considered statistically significant. *p < 0.05; **p < 0.01; ***p < 0.001.

## 3 Results

### 3.1 Characteristics of circFOXK2 in NSCLC Cells

**FIGURE 1:**
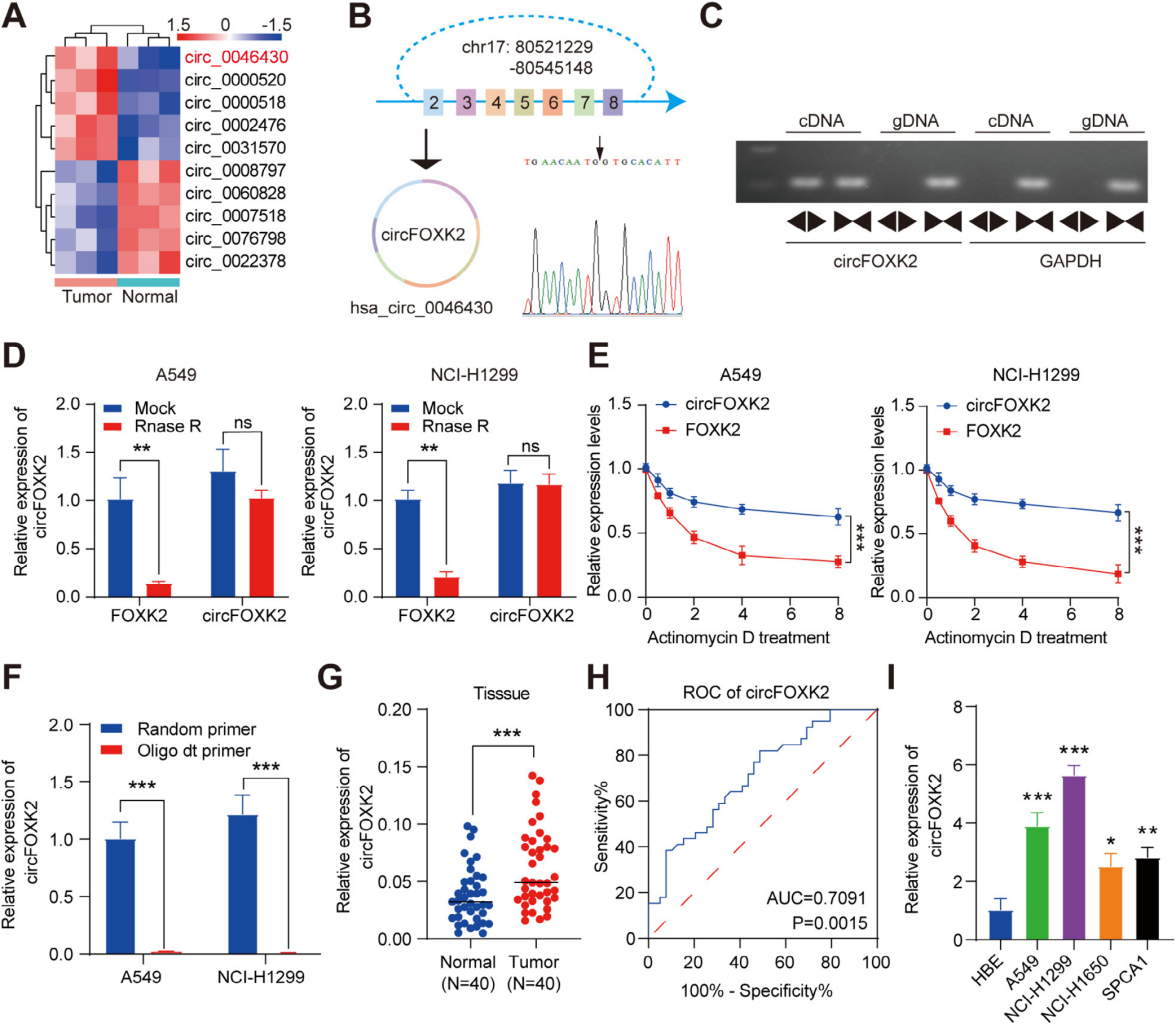
CircFOXK2 is upregulated in NSCLC and is associated with progression. (A) Clustering shows the expression changes of circular RNAs compared to adjacent normal tissues in three cases of NSCLC tissues. (B) The structure of circFOXK2 was confirmed by Sanger sequencing. (C) Analysis of circFOXK2 and GAPDH products amplified with convergent and divergent primers by PCR and agarose gel electrophoresis, respectively. (D, E) Expression changes of circFOXK2, linear FOXK2, and GAPDH after treatment with RNase R and Actinomycin D, as detected by qRT‐PCR. (F) qRT‐PCR detects the expression changes of circFOXK2 in A549 and NCI‐H1299 cells. (G) qRT‐PCR analysis of the relative expression of circFOXK2 in 40 pairs of NSCLC tissues. (H) ROC curve analysis of the diagnostic value of circPTK2 in 40 pairs of NSCLC tissues (AUC = 0.7091, *p* < 0.0015). (I) qRT‐PCR assessment of circFOXK2 expression levels in HBE, A549, NCI‐H1299, NCI‐1650, and SPC‐A1 cells. *p < 0.05; **p < 0.01; ***p < 0.001.

Li et al. performed circRNA profiling on three pairs of NSCLC tissues to identify circRNAs with differential expression in NSCLC (the screening criteria were |log 2 FC| > 1, FDR < 0.05) [30]. A total of 109 circRNAs were identified as differentially expressed, including 33 that were upregulated and 76 that were downregulated. Among them, we focused on hsa_circ_0046430, which was detected in all tissues and exhibited a significant fold change (log 2 FC = 3.2470275) (Figure 1A). Initially, specific primers were designed for qRT‐PCR to characterize the circRNA. Sanger sequencing of the amplified products revealed a back‐splicing event at the junction of exons 2–8 of FOXK2 (Figure 1B). We designated this novel circRNA as “circFOXK2” and confirmed its characteristic circular RNA features. Using divergent primers (spanning the back‐splice junction) and convergent primers, we tested the head‐to‐tail splicing of endogenous circFOXK2. In this regard, circFOXK2 could be amplified specifically from cDNA using different primers but not from genomic DNA (gDNA) (Figure 1C). To assess circFOXK2's stability, treatments with RNase R and actinomycin D were performed. The results indicated that RNase R effectively degraded linear transcripts, whereas circular transcripts remained resistant to digestion by RNase R (Figure 1D), thus demonstrating the resistance of circFOXK2 to RNase R degradation. Additionally, after exposure to actinomycin D, circFOXK2 demonstrated increased stability compared to its linear counterpart (Figure 1E). Furthermore, compared to oligo(dT) primers, circFOXK2 showed superior reverse transcription efficiency with random primers (Figure 1F). This finding suggests that circFOXK2 lacks a polyadenylated tail, a characteristic feature of circular RNA. In conclusion, these findings indicate that circFOXK2 is a bona fide circRNA with the expected molecular structure and biochemical properties.

To further validate the abnormal expression of circFOXK2 in NSCLC tissues, qRT‐PCR was performed on 40 matched pairs of NSCLC and adjacent normal tissues. Our results demonstrate that circFOXK2 is upregulated in NSCLC tissues compared to normal tissues (Figure 1G), confirming the RNA sequencing findings. The ROC curve demonstrated that circFOXK2 had diagnostic value in NSCLC tissues, with an AUC value of 0.7091 (Figure 1H). Additionally, upregulation of circFOXK2 levels was observed in NSCLC cell lines (A549, NCI‐H1299, SPC‐A1, and NCI‐H1650) compared to bronchial epithelial cells (HBE) (Figure 1I). We proceeded with the two cell lines exhibiting the highest expression levels (A549 and NCI‐H1299) for further experiments.

### 3.2 circFOXK2 Acts as an Oncogene and Accelerates the Progression of NSCLC

**FIGURE 2:**
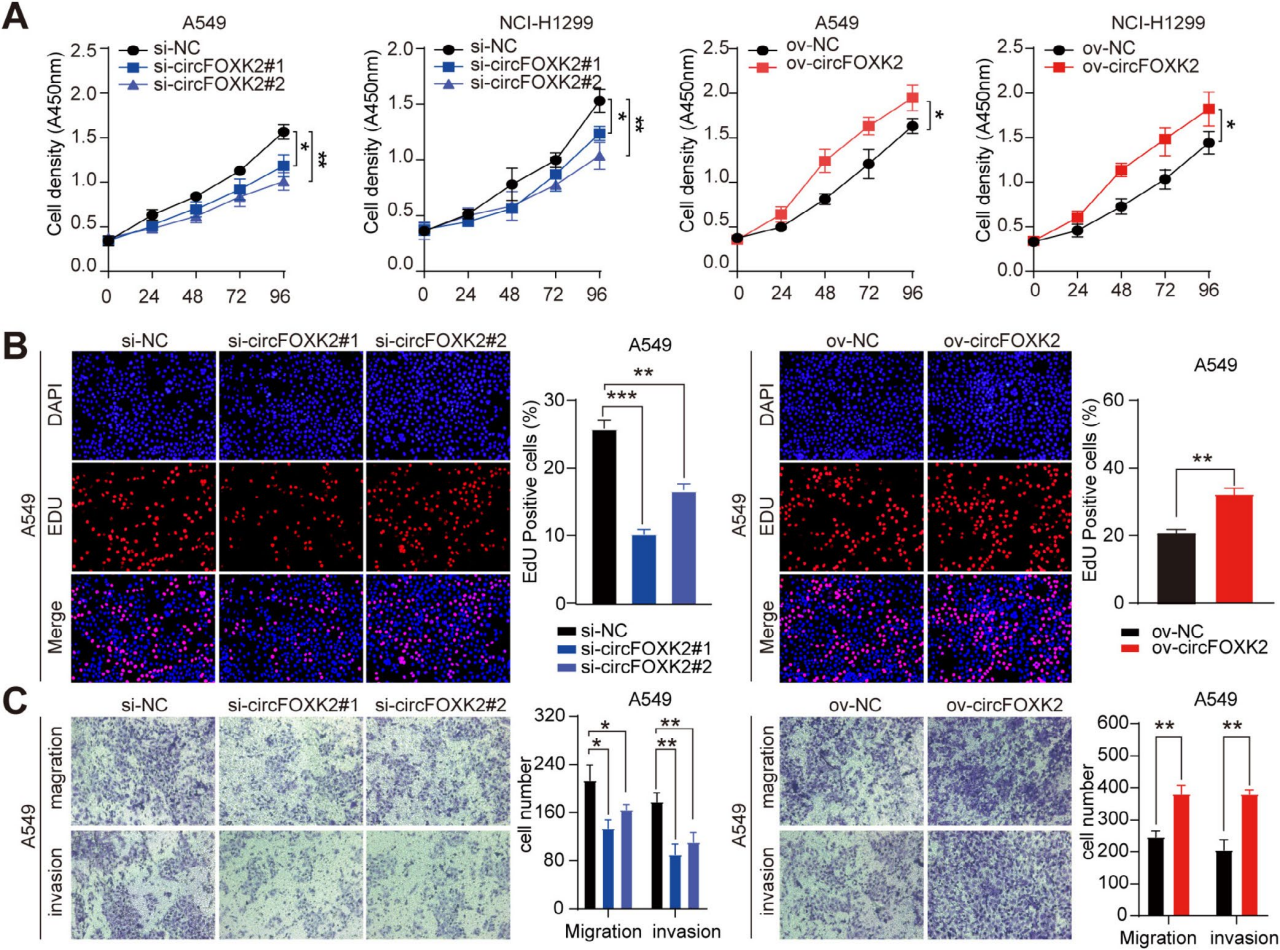
CircFOXK2 promotes NSCLC cells proliferation, migration, and invasion in vitro. (A) The growth curves of A549 and NCI‐H1299 cells after knockdown or overexpression of circFOXK2 were evaluated using the CCK‐8 assay. (B) To investigate the effect of circFOXK2 on the proliferation of A549 cells, an EdU assay was performed, scale bar = 50 μm. (C) Transwell assays were conducted on A549 cells to detect migration and invasion abilities after knockdown or overexpression of circFOXK2. *p<0.05; **p<0.01; ***p<0.001.

To explore the role of circFOXK2 in NSCLC progression, we constructed a circFOXK2 overexpression plasmid and confirmed the accurate and efficient overexpression of circFOXK2 in NSCLC cells. To knock down circFOXK2, we designed three specific siRNAs targeting the back‐splice junction region. si‐circFOXK2 #1 and si‐circFOXK2 #2 successfully silenced circFOXK2 (Figure S1A). Our subsequent aim was to evaluate the role of circFOXK2 in the proliferation, invasion, and migration of NSCLC cells. CCK and EDU assays showed that silencing circFOXK2 inhibited the proliferative capacity of A549 and NCI‐H1299, while overexpression yielded opposite results (Figure 2A,B and Figure S1B). Transwell assays demonstrated that silencing circFOXK2 inhibited the migration and invasion capacities of A549 and NCI‐H1299 cells, while overexpression significantly enhanced these abilities in NSCLC cells, as expected (Figure 2C and Figure S1C). In conclusion, our results strongly suggest that upregulated circFOXK2 acts as an oncogene, accelerating NSCLC progression.

### 3.3 STMN1 Is an Important Factor Mediated by circFOXK2 in the Malignancy of NSCLC

**FIGURE 3:**
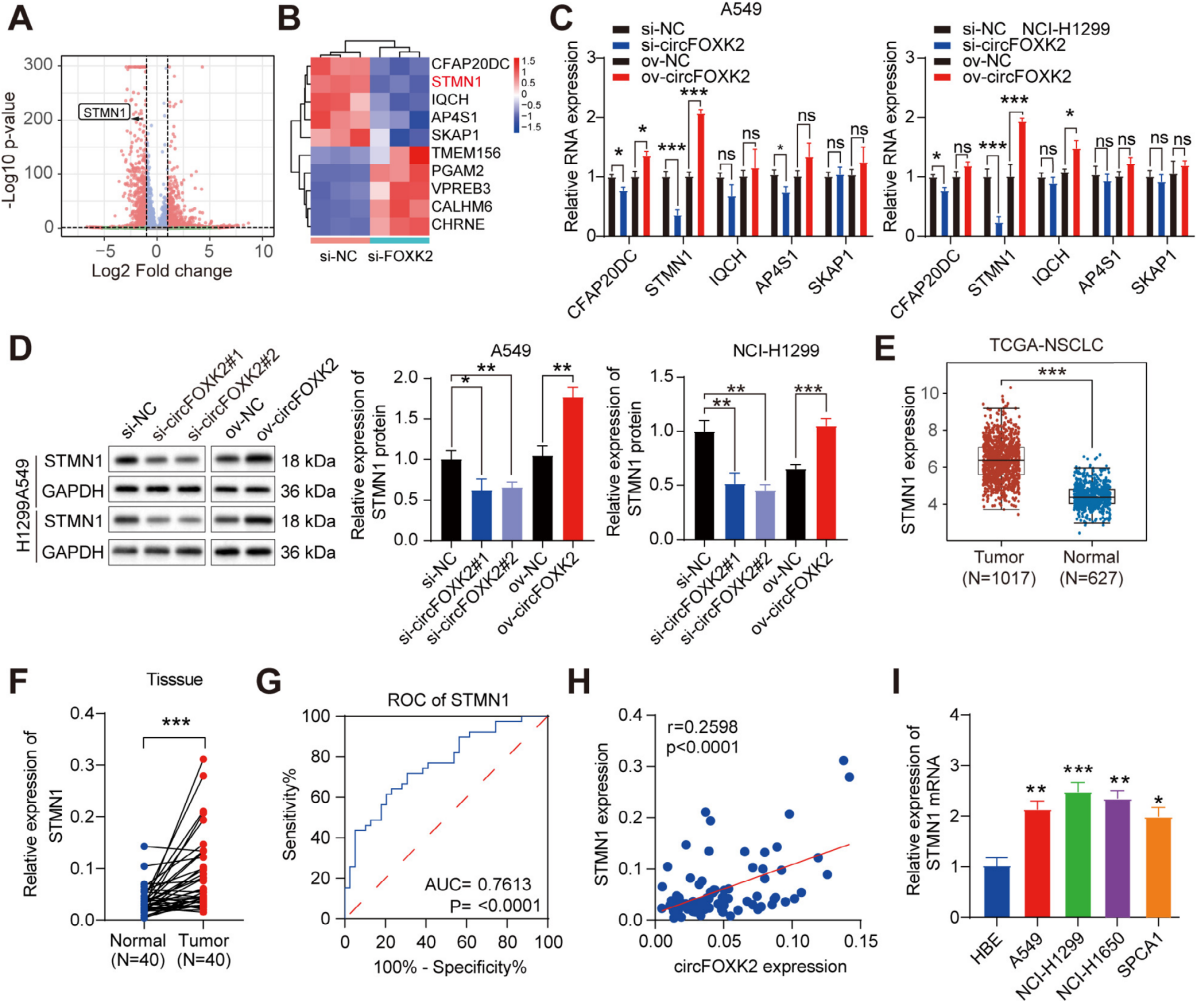
STMN1 is an important factor mediated by circFOXK2 in the malignancy of NSCLC. (A,B) Clustered heat maps and volcano plots show the expression changes of mRNA after silencing circFOXK2 compared to the control group. (C) After circFOXK2 knockdown or overexpression, STMN1, IQCH, AP4S1, SKAP1, and CFAP20DC expression changes were detected by qRT‐PCR. (D) Western blot analysis of STMN1 protein expression changes after circFOXK2 knockdown or overexpression. (E) STMN1 expression in NSCLC tissues from the TCGA database. (F) qRT‐PCR of STMN1 relative expression in 40 pairs of NSCLC samples. (G) ROC curve analysis for the diagnostic value of STMN1 in 40 pairs of NSCLC tissues (AUC = 0.7613, *p* < 0.0001). (H) Correlation between circFOXK2 and STMN1 verified in 40 NSCLC tissues. (I) qRT‐PCR and Western blot analysis of STMN1 expression levels in HBE, A549, NCI‐H1299, and SPC‐A1 cells. *p < 0.05; **p < 0.01; ***p < 0.001.

To uncover the specific mechanism by which circFOXK2 exerts its oncogenic function, we performed RNA‐Seq to screen the transcriptome, focusing on genes with decreased expression in the cells when circFOXK2 was knocked down compared to control cells (Figure 2A). The results revealed a total of 1481 differentially expressed genes, including 765 upregulated circRNAs and 716 downregulated differentially expressed genes (Figure 3A,B). Subsequently, we selected the top 5 significantly different candidate genes for further investigation. We found that STMN1 was downregulated when circFOXK2 was silenced, while it was upregulated with the overexpression of circFOXK2 (Figure 3C). Furthermore, the alterations in STMN1 protein levels in NSCLC cells following the modulation of circFOXK2 were validated by Western blot analysis (Figure 3D). Overall, we confirmed STMN1 as a potential downstream target of circFOXK2, worthy of further investigation.

To explore the potential involvement of STMN1 in NSCLC, we evaluated its aberrant expression using public databases and 40 pairs of clinical samples. The results indicated upregulation of STMN1 in NSCLC, which distinguished tumor tissue from normal tissue (Figure 3E,F). The ROC curve demonstrated that STMN1 had diagnostic value in NSCLC tissues, with an AUC value of 0.7613 (Figure 3G). However, no significant difference in survival outcomes was observed between the groups with high and low STMN1 expression (Figure S2A). In clinical samples, the expression of circFOXK2 showed a positive correlation with STMN1 (Figure 3H). Additionally, elevated levels of STMN1 were observed in NSCLC cell lines (A549, NCI‐H1299, SPC‐A1, and NCI‐H1650) compared to the bronchial epithelial cell line (HBE) (Figure 3I), suggesting a potential oncogenic role for abnormal STMN1 in NSCLC.

**FIGURE 4:**
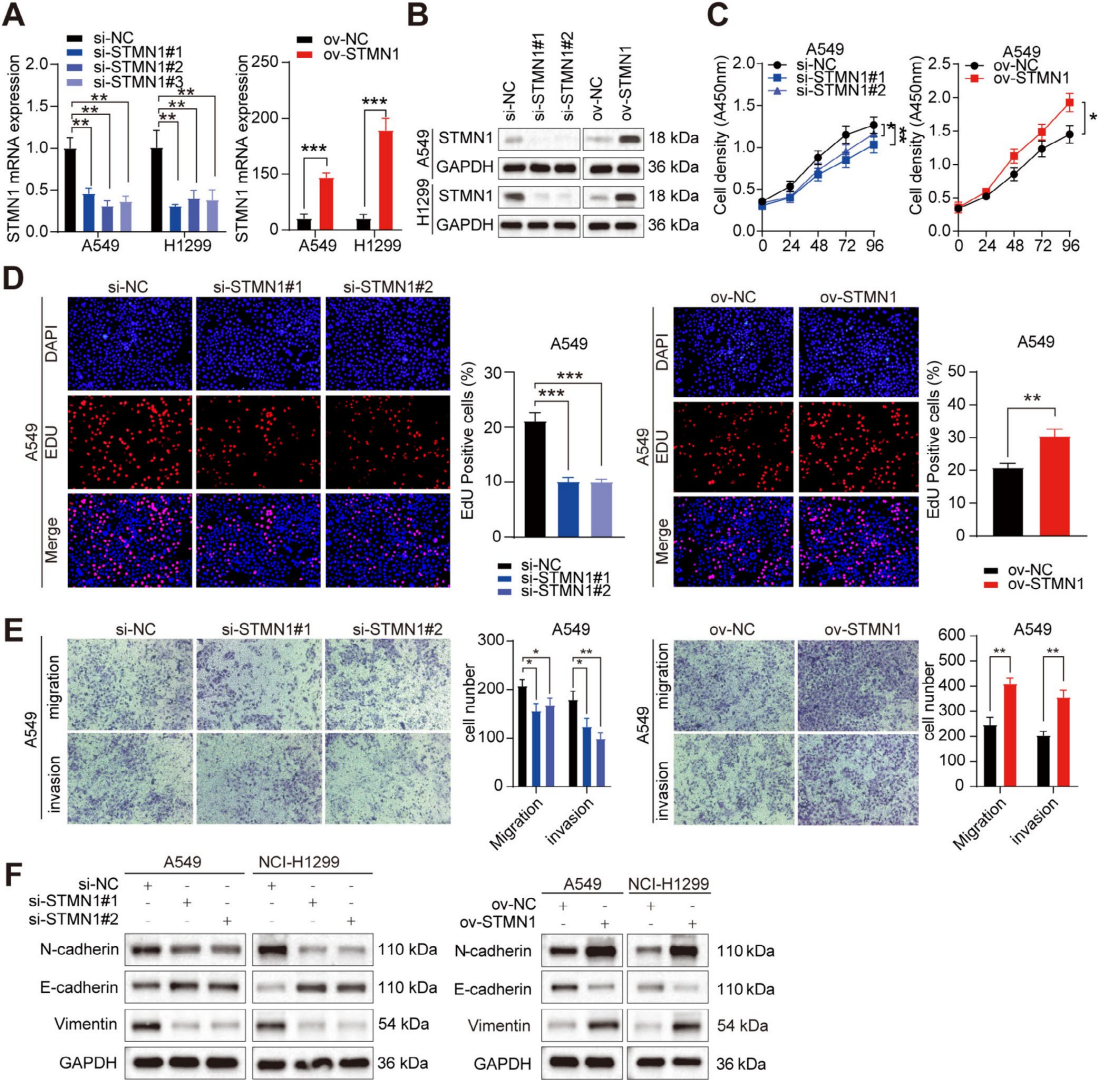
STMN1 promotes NSCLC cells proliferation, migration, and invasion in vitro. (A) Relative expression of STMN1 in A549 and NCI‐H1299 cells transfected with STMN1 overexpression plasmid or siRNA. (B) The expression of STMN1 was detected by Western blot after knockdown or overexpression. (C) The growth curve of A549 cells was evaluated with CCK‐8 assay after STMN1 knockdown or overexpression. (D) The effect of STMN1 on the proliferation of A549 cells was assessed using the EdU assay, with a scale bar of 50 μm. (E) The migration and invasion capabilities after STMN1 knockdown or overexpression in A549 cells were examined by Transwell assay. (F) After STMN1 knockdown or overexpression, Western blot was used to detect the expression of EMT‐related proteins in A549 and NCI‐H1299 cells. *p < 0.05; **p < 0.01; ***p < 0.001.

Following this, to study the potential biological role of STMN1 in NSCLC cells, we transfected A549 and NCI‐H1299 cells with plasmids targeting STMN1 and three independent siRNAs (Figure 4A,B). CCK and EDU assays revealed that silencing STMN1 inhibited the proliferative capacity of A549 and NCI‐H1299 cells, while overexpression of STMN1 yielded the opposite results (Figure 4C,D and Figure S2B,C). Transwell assays demonstrated that silencing STMN1 inhibited the migration and invasion abilities of A549 and NCI‐H1299 cells, while overexpression of STMN1 significantly accelerated the invasion and migration of NSCLC cells (Figure 4E and Figure S2D). Considering the significant influence of the circFOXK2‐STMN1 axis on NSCLC biological behavior, we attempted to elucidate its specific mechanism and biological impacts. EMT is considered a key driving factor in tumor invasion and metastasis. In our study, we found that overexpression of STMN1 led to increased expression of mesenchymal markers (N‐cadherin, vimentin), while the epithelial marker (E‐cadherin) was decreased, and silencing of STMN1 produced the opposite effect (Figure 4F), suggesting an induction of EMT by STMN1 in NSCLC cells.

**FIGURE 5:**
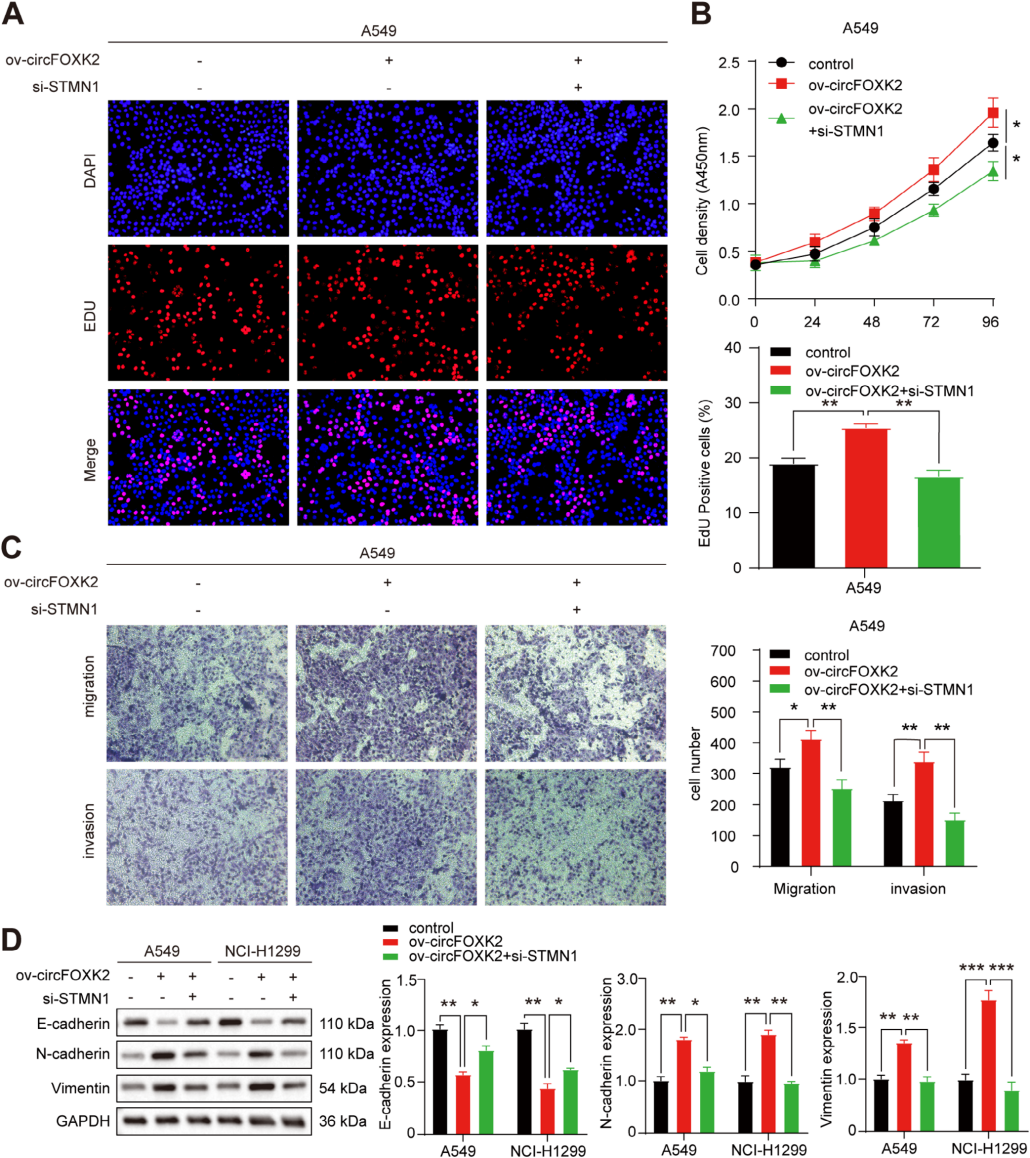
CircFOXK2 promotes the proliferation and metastasis of NSCLC by regulating STMN1. (A, B) EdU incorporation and CCK‐8 assays showed that co‐transfection of circFOXK2 overexpression plasmid and si‐STMN1 in A549 cells can counteract the promotion effect caused by circFOXK2 overexpression. (C) Transwell assay in A549 cells indicates that STMN1 knockdown recovers the promotion effect of circFOXK2 overexpression on migration and invasion. (D) Western blot results indicate that STMN1 reverses the effect of circTHBS1 overexpression on the expression of key proteins involved in EMT. *p < 0.05; **p < 0.01; ***p < 0.001.

We performed rescue experiments to investigate whether circFOXK2 promotes tumorigenesis and malignant transformation through STMN1 mediation. CCK and EDU assays showed that partial knockdown of STMN1 reduced the proliferative effects of circFOXK2 overexpression in NSCLC cells compared to controls (Figure 5A,B and Figure S3A,B). Transwell analysis showed that partial knockdown of STMN1 reduced the effects of circFOXK2 overexpression on the migration and invasion of NSCLC cells compared to controls (Figure 5C and Figure S3C). Furthermore, overexpression of circFOXK2 resulted in a significant decrease in E‐cadherin levels and increased mesenchymal markers. These changes were abolished by silencing STMN1 (Figure 5D).

### 3.4 The Interaction Between circFOXK2 and the PABPC1 Protein in NSCLC Cells

**FIGURE 6:**
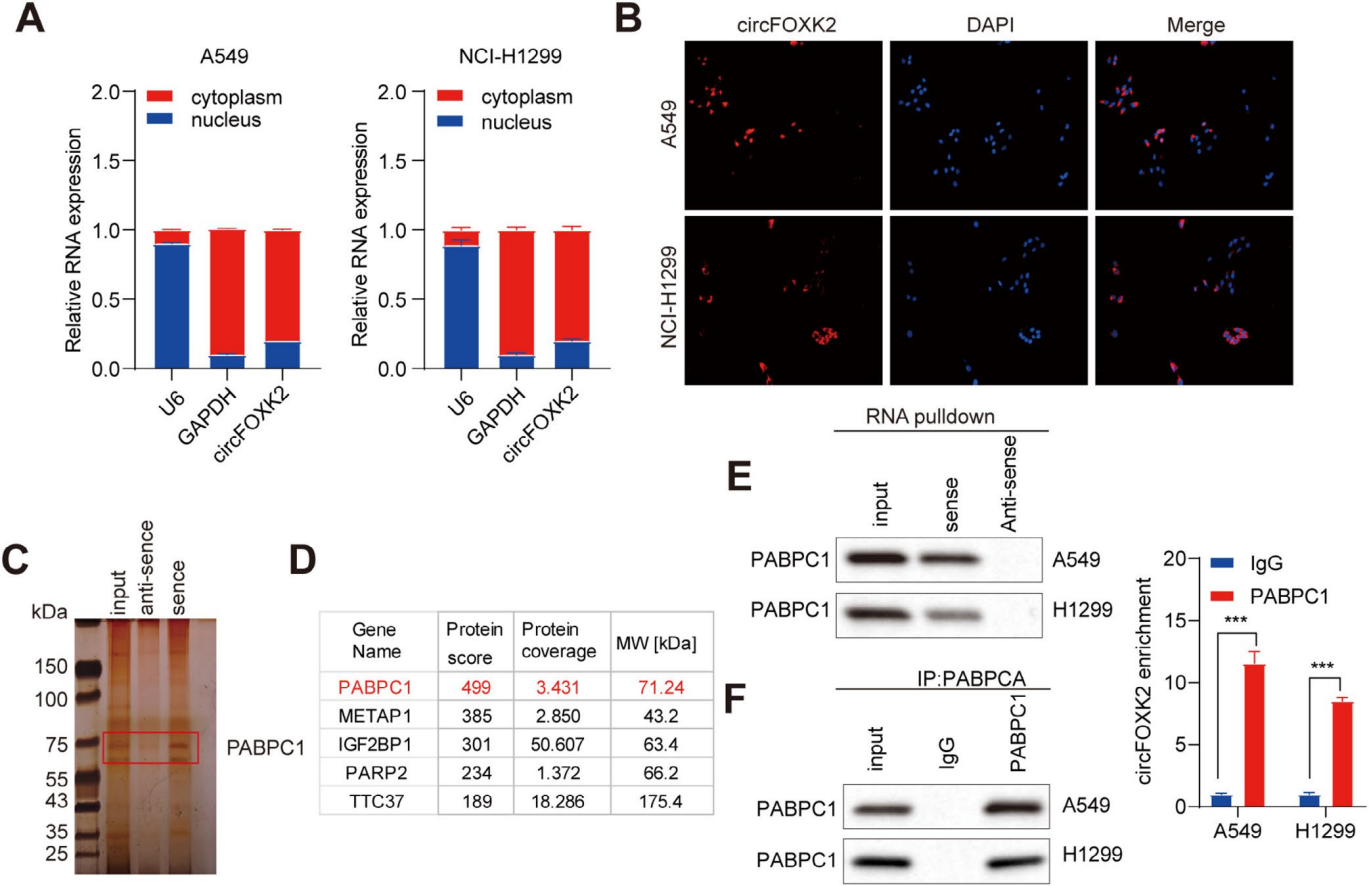
The interaction between circFOXK2 and the PABPC1 protein in NSCLC cells. (A) qRT‐PCR results of nuclear‐cytoplasmic separation indicate that circFOXK2 is mainly localized in the cytoplasm in A549 and NCI‐H1299 cell lines. (B) FISH of circFOXK2. The cell nucleus is stained with DAPI. Scale bar = 50 μm. (C, D) Silver staining and mass spectrometry identify proteins bound to biotinylated circFOXK2. (E) RNA pull‐down assay detects the interaction between circFOXK2 and PABPC1 in A549 and NCI‐H1299 cells. (F) RIP assay detects the interaction between endogenous PABPC1 and circFOXK2 in A549 and NCI‐H1299 cells. ***p < 0.001.

The subcellular localization of circFOXK2 is crucial for a better understanding of its biological roles. To probe how circFOXK2 regulates STMN1 expression, we first conducted qRT‐PCR on separated nuclear and cytoplasmic fractions. The findings demonstrated that circFOXK2 is predominantly localized in the cytoplasmic fraction of NSCLC cells (Figure 6A). Moreover, fluorescence in situ hybridization corroborated this, showing the main distribution of circFOXK2 in the cytoplasm (Figure 6B). We speculated whether circFOXK2 acts as a miRNA sponge to inhibit NSCLC progression. Nonetheless, RNA immunoprecipitation assays revealed that circFOXK2 did not bind to AGO2 (Figure S4A). Therefore, we excluded the possibility of circFOXK2 acting as a miRNA sponge.

Investigating how circRNA interacts with RNA‐binding proteins (RBPs) can unveil the core mechanisms underpinning their functionality [31]. In our research, we used a biotin‐labeled circFOXK2 probe and mass spectrometry analysis in RNA pulldown assays to screen for circFOXK2‐interacting proteins (Figure 6C). Mass spectrometry analysis found no evidence of an interaction between circFOXK2 and AGO2, which aligns with our previous findings from the RIP assays. PABPC1 emerged from the mass spectrometry data as the top‐scoring or uniquely identified circFOXK2‐binding protein (Figure 6D). Therefore, we determined that PABPC1 is a putative circFOXK2‐binding protein. Through RNA pulldown experiments, we observed specific precipitation of PABPC1 in the circFOXK2 pulldown (Figure 6E). Moreover, compared to the control IgG, RIP experiments demonstrated that circFOXK2 was more enriched in complexes precipitated with the anti‐PABPC1 antibody (Figure 6F). Taken together, these results suggest that circFOXK2 mediates its pathological effects in NSCLC cells by directly interacting with PABPC1 to form a circRNA‐protein complex.

### 3.5 circFOXK2/PABPC1 Complex Stabilizes STMN1 mRNA

**FIGURE 7:**
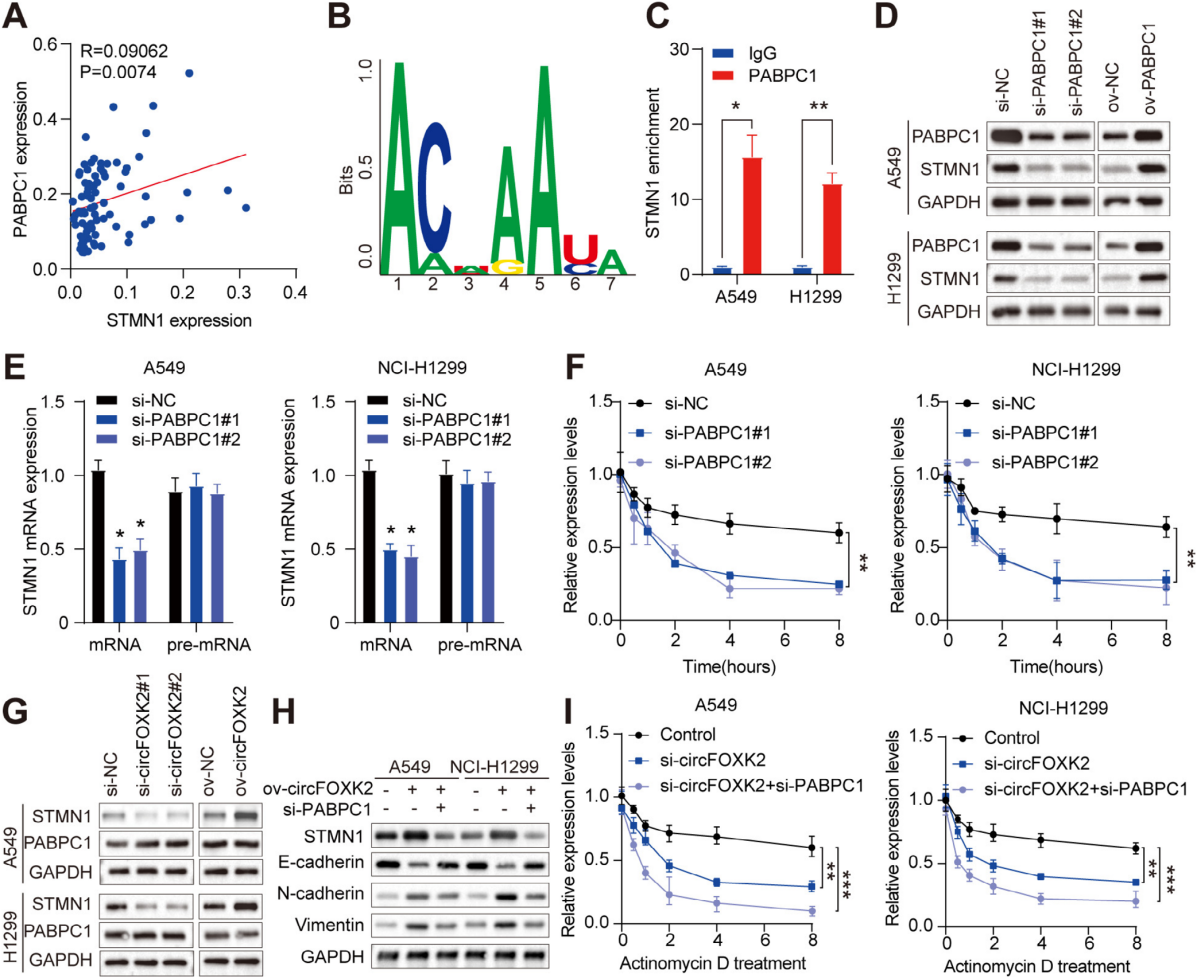
circFOXK2/PABPC1 complex stabilizes STMN1 mRNA. (A) Verification of STMN1 correlation with PABPC1 in 40 NSCLC tissues. (B) Bioinformatics analysis of potential PABPC1 binding motifs. (C) RIP assay detects the interaction between endogenous STMN1 and circFOXK2 in A549 and NCI‐H1299 cells. (D) Western blot analysis of PABPC1 protein expression after overexpression or knockdown. (E) qRT‐PCR was used to detect STMN1 mRNA and pre‐mRNA levels following PABPC1 silencing. (F) After silencing of PABPC1, STMN1 expression levels were detected by qRT‐PCR following treatment with Actinomycin D. (G, H) Western blot analysis of PABPC1 and STMN1 protein expression after circFOXK2 overexpression or knockdown. (I) After treatment with Actinomycin D, qRT‐PCR detects the expression levels of STMN1. *p < 0.05; **p < 0.01; ***p < 0.001.

PABPC1 is a classic polyadenylate‐binding protein that regulates the stability of mRNA through binding to the poly(A) tail or the 5′‐ACUAAUC‐3′ motif of mRNA [32, 33]. Given the critical role of PABPC1 in mRNA stability, we aimed to investigate whether circFOXK2 promotes mRNA stability by interacting with PABPC1, thereby affecting STMN1 expression and driving tumor progression. To evaluate this situation, we initially observed the upregulation of PABPC1 in clinical samples of NSCLC tumors (Figure S4B). No statistically significant discrepancy was observed in circFOXK2 and PABPC1 expression levels (Figure S4C), and in clinical samples, a strong correlation between PABPC1 and STMN1 was validated (Figure 7A). Furthermore, we used the AURA database (http://aura.science.unitn.it//.,m) to forecast the binding motifs of PABPC1 and identify potential mRNA targets for PABPC1. It was surprising to find a potential binding relationship between STMN1 3′‐UTR and PABPC1 (Figure 7B). RIP assays showed enrichment of STMN1 in the complex precipitated with anti‐PABPC1 antibody, in contrast to the IgG control (Figure 7C). Subsequently, we silenced PABPC1 in cells to assess its impact on STMN1 expression. Following PABPC1 knockdown, both STMN1 protein and mRNA expression decreased (Figure 7D). However, we observed no change in pre‐mRNA expression of STMN1 after PABPC1 siRNA treatment, indicating that PABPC1 does not influence the synthesis of new STMN1 mRNA (Figure 7E). Therefore, we evaluated the stability of STMN1 mRNA after altering PABPC1. Interestingly, using actinomycin D to block new RNA synthesis, RNA decay assays showed a significant decrease in the half‐life of STMN1 mRNA after PABPC1 silencing (Figure 7F). Taken together, the results indicate that PABPC1 can bind to STMN1 mRNA, acting as an mRNA stabilizer in NSCLC, thereby delaying its degradation.

We then described whether circFOXK2 exerts oncogenic effects in NSCLC through PABPC1. Through Western blot and qRT‐PCR, we first ruled out the regulatory impact of circFOXK2 on PABPC1 expression. The findings indicated that silencing circFOXK2 indeed inhibited STMN1 expression, while PABPC1 expression remained unaffected. This was in line with earlier RNA‐Seq results. Additionally, overexpression of circFOXK2 did not elevate PABPC1 levels (Figure 7G). Subsequently, we conducted PABPC1 knockdown experiments in NSCLC cells with stable high expression of circFOXK2. Protein blot analysis indicated that overexpression of circFOXK2 elevated the level of STMN1. Still, this effect was nullified when PABPC1 was knocked down (Figure 7H), suggesting that circFOXK2 enhances STMN1 expression in vitro in a manner reliant on PABPC1. Furthermore, RNA decay assays indicated that the silencing of circFOXK2 led to a shortened half‐life of STMN1 mRNA (Figure 7I). Importantly, the concurrent silencing of circFOXK2 and PABPC1 further increased the instability of STMN1 mRNA, indicating a synergistic action of PABPC1 and circFOXK2 in maintaining the stability of STMN1 mRNA. In summary, our results indicate that the circFOXK2/PABPC1 complex can stabilize STMN1 mRNA in NSCLC. Furthermore, we found that knocking down PABPC1 negated the promotion of EMT by circFOXK2 (Figure 7H), suggesting that PABPC1 induces EMT in NSCLC cells.

### 3.6 circFOXK2 Promotes Growth and Metastasis of NSCLC In Vivo

**FIGURE 8:**
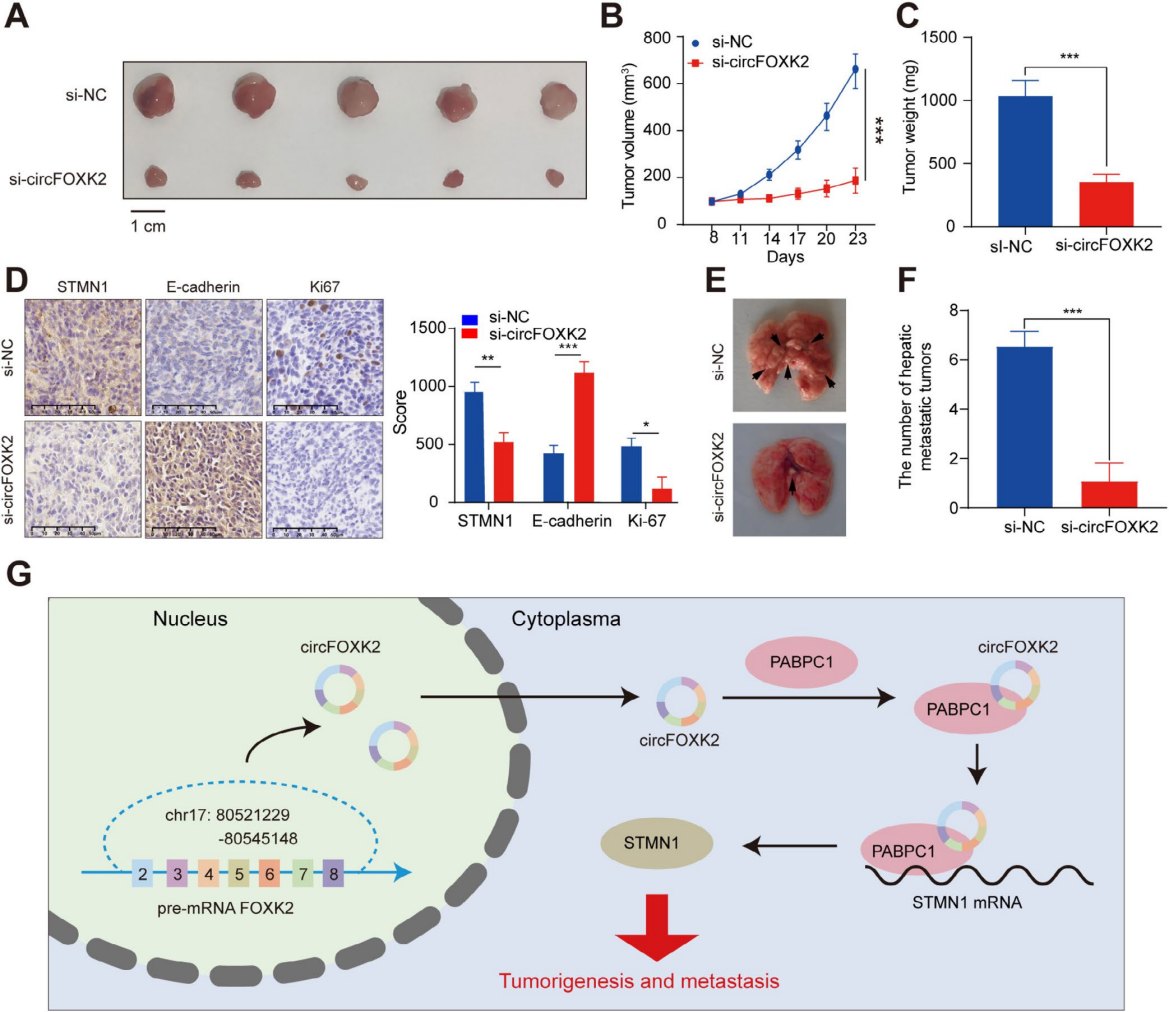
circFOXK2 promotes growth and metastasis of NSCLC in vivo. (A, B) Subcutaneous tumor growth curves in mice (*n* = 6), with volume measured every 3 days. (C) Four weeks after subcutaneous injection of A549 cells with stable knockdown of circFOXK2, mice were euthanized, and the volume and weight of ectopic transplants were measured. (D) Immunohistochemical staining of Ki67, E‐cadherin, and STMN1 in ectopic transplants. (E) Schematic representation of metastatic nodules in the lungs 4 weeks after mice were injected with A549 cells with stable knockdown of circFOXK2 via tail vein. (F) H&E staining of metastasis in nude mouse lungs. (G) Proposed model illustrating the mechanism of circFOXK2/PABPC1/STMN1 axis in NSCLC. *p < 0.05; **p < 0.01; ***p < 0.001.

To assess the biological role of circFOXK2 in vivo, we subcutaneously injected A549 cells with stable knockdown of circFOXK2 into the flank of nude mice. The subcutaneous tumors were monitored once a week (Figure 8A,B) and collected in the fourth week. silencing of circFOXK2 reduced the weight of the tumors compared with the control group (Figure 8C). Immunohistochemical staining for Ki67, E‐cadherin, and STMN1 showed significantly reduced expression in the circFOXK2 silenced group (Figure 8D). We further investigated the impact of circFOXK2 on tumor metastasis. The findings indicated that silencing of circFOXK2 significantly inhibited lung metastasis compared to the control group (Figure 8E,F). From these findings, we conclude that circFOXK2 facilitates the proliferation and metastasis of NSCLC in vivo.

In conclusion, these findings suggest that upregulated circFOXK2 can bind to PABPC1 and enhance the stability of STMN1 mRNA, thereby promoting the proliferation and metastasis of NSCLC (Figure 8G).

## 4 Discussion

With advancements in high‐throughput sequencing and bioinformatics technologies, circRNAs are no longer considered byproducts of mRNA splicing but are recognized to possess essential regulatory functions [34]. A growing number of circRNAs are continuously being screened and validated for their expression and functions, and they are believed to participate in various biological differentiation and developmental processes in a highly regulated manner [35, 36]. Furthermore, a large number of dysregulated circRNA expressions have been implicated in the initiation and progression of cancer [37]. Then, our research revealed significantly elevated expression of circFOXK2 in NSCLC using bioinformatics methods. Additionally, we have validated through tissue analysis the high expression of circFOXK2 in tumor tissues, indicating its ongoing diagnostic value as a biomarker for NSCLC.

We further analyzed the specific molecular mechanisms of circFOXK2 in NSCLC. Our experimental results demonstrate that circFOXK2 promotes the proliferation, migration, and invasion abilities of lung cancer cell lines. Apart from promoting the progression of NSCLC, STMN1 is also a poor prognostic biomarker for NSCLC [38]. STMN1 can enhance the radioresistance of tumor cells by increasing autophagy [39], and it can mediate drug resistance in tumors through the AKT and EGFR‐related pathways [40, 41]. Interestingly, in our transcriptome analysis of circFOXK2 knockdown, we observed significant downregulation of STMN1. Therefore, we investigated whether circFOXK2's regulatory effect on NSCLC is related to STMN1. Our dual treatment experiment involving circFOXK2 and STMN1 revealed a close relationship between the two in NSCLC. In summary, the promotive effect of circFOXK2 on NSCLC development depends on the expression of STMN1.

circRNAs have roles in sponging miRNA [35, 42]. In this research, we demonstrate that circFOXK2 exerts its effects not via the ceRNA mechanism, but rather by interacting with the PABPC1 protein to influence the stability of STMN1 mRNA. Initially isolated and cloned from human melanoma cells, PABPC1 is evolutionarily conserved throughout eukaryotes [43]. PABPC1 contains four RNA recognition motif (RRM) domains capable of RNA binding, while its C‐terminal MLLE domain mediates binding to peptide motifs such as PAM2, allowing PABPC1 to interact with numerous proteins including PAIP1, PAIP2, GW182, and MKRN1 [44, 45, 46]. Comparison of the Cancer Genome Atlas (TCGA) and Genotype‐Tissue Expression (GTEx) datasets with normal tissues showed notable upregulation of PABPC1 in 31 tumor samples. Notably, PABPC1 is crucial for maintaining mRNA stability, particularly by safeguarding long poly(A) tails from uridylation, a specificity likely induced by its length‐dependent binding [47]. Previous studies have indicated that in esophageal squamous cell carcinoma, PABPC1 fosters both angiogenesis and malignant progression by inducing IFI27 mRNA stability [48], and PABPC1 de‐ubiquitinated by USP10 promotes the stability of CLK2 mRNA to facilitate the development of pancreatic cancer [49]. Here, we reveal a similar role. Through co‐immunoprecipitation and mass spectrometry experiments, we found that circFOXK2 interacts with PABPC1, and the circFOXK2/PABPC1 complex maintains the stability of STMN1 mRNA, promoting the process of tumor epithelial–mesenchymal transition (EMT).

Although we have elucidated the mechanism through which circFOXK2 regulates STMN1 via PABPC1, numerous specific mechanisms remain to be investigated. PABPC1 has the ability to bind to a large number of RNAs and proteins, and more experiments are needed to authenticate the interaction between circFOXK2 and PABPC1 definitively. Additionally, we are keenly interested in determining whether other RNAs and proteins are necessary for the function of the circFOXK2/PABPC1 complex. Furthermore, the maintenance of STMN1 mRNA stability by the circFOXK2/PABPC1 complex represents a particularly intriguing mechanism. The interaction patterns and dependency recognition of PABPC1 with STMN1 mRNA are still to be explored.

In summary, through in vitro and in vivo experiments, we have elucidated the interaction between circFOXK2 and PABPC1, and how the circFOXK2/PABPC1 complex maintains the stability of STMN1 mRNA, thereby promoting the occurrence and development of NSCLC.

## Author Contributions

Data collecting: W.C., X.W., T.F., S.F., and C.Z. Writing: W.C., Z.C., and S.F. Data analysis: W.C., Z.C., W.S., X.Z., Y.H., and Y.X. Design: C.Z. and S.F. All authors read and approved the final manuscript.

## Ethics Statement

All animal experiments and procedures received approval and were conducted in accordance with the Animal Experimentation Ethics Committee of Ningbo University School of Medicine. This study was approved and performed by the Ethics Committee of the First Affiliated Hospital of Ningbo University. The tumors in the experimental mice did not exceed the maximum tumor burden.

## Conflicts of Interest

The authors declare no conflicts of interest.

## Supporting information


**Figure S1.** circFOXK2 promotes NSCLC cells proliferation, migration, and invasion in vitro. (A) The relative expression of circFOXK2 in A549 and NCI‐H1299 cells transfected with circFOXK2 overexpression plasmid or siRNA. (B) The impact of circFOXK2 on the proliferation of NCI‐H1299 cells was evaluated using the EdU assay, scale bar = 50 μm. (C) Transwell assays were performed on NCI‐H1299 cells to examine the migration and invasion abilities after knockdown or overexpression of circFOXK2. *p<0.05; **p<0.01; ***p<0.001.

**Figure S2.** STMN1 promotes NSCLC cells proliferation, migration, and invasion in vitro. (A) Survival analysis of NSCLC patients based on STMN1 expression in the TCGA database. (B) The growth curve of NCI‐H1299 cells was evaluated using the CCK‐8 assay after STMN1 knockdown or overexpression. (C) The impact of STMN1 on the proliferation of NCI‐H1299 cells was evaluated using the EdU assay, with a scale bar of 50 μm. (D) The migration and invasion capabilities of NCI‐H1299 cells after STMN1 knockdown or overexpression were examined by Transwell assay. *p<0.05; **p<0.01; ***p<0.001.

**Figure S3.** CircFOXK2 promotes the proliferation and metastasis of NSCLC by regulating STMN1. (A, C) EdU assays and CCK‐8 experiments indicated that co‐transfection with circFOXK2 overexpression plasmid and si‐STMN1 in NCI‐H1299 cells could offset the promotional effect caused by the overexpression of circFOXK2. (B) Transwell assays of NCI‐H1299 cells demonstrated that STMN1 knockdown restored the enhanced effects on migration and invasion due to overexpression of circFOXK2. *p<0.05; **p<0.01; ***p<0.001.

**Figure S4.** (A) RIP assay was used to detect the interaction between AGO2 and circFOXK2 in A549 and NCI‐H1299 cells. (B) qRT‐PCR was employed to measure the relative expression of PABPC1 in 40 pairs of NSCLC tissues. (C) The correlation between circFOXK2 and PABPC1 was validated in 40 cases of NSCLC tissues. *p<0.05; **p<0.01; ***p<0.001

**Table S1.** Primer sequences.
**Table S2.** RNA oligonucleotide sequences.

## Data Availability

STMN1 and PABPC1 mRNA expression profiles in NSCLC were obtained from the TCGA database. The direct interaction between PABPC1 and circFOXK2 was drawn using the RNA Binding Protein Database (RBPDB; http://rbpdb.ccbr.utoronto.ca/).
