# Supplementary figures and images for: circFOXK2 Stabilizes STMN1 mRNA via PABPC1 to Promote the Progression of NSCLC
Source: Cancer Med. 2025 Feb 27;14(5):e70729. doi: 10.1002/cam4.70729 (PMC11866311; doi:10.1002/cam4.70729)

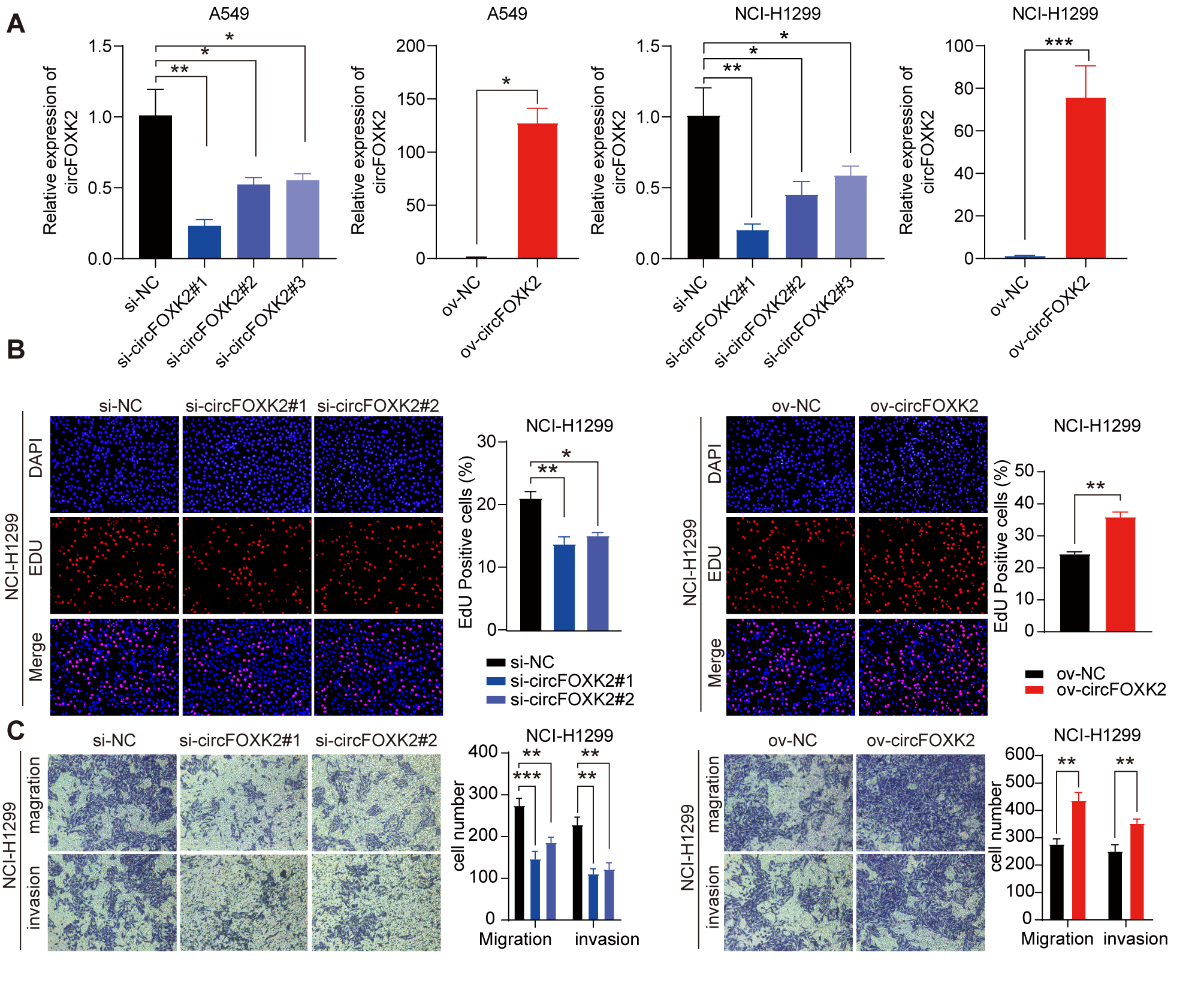

Supplement: Supplementary file 1 — Figure S1. circFOXK2 promotes NSCLC cells proliferation, migration, and invasion in vitro. (A) The relative expression of circFOXK2 in A549 and NCI‐H1299 cells transfected with circFOXK2 overexpression plasmid or siRNA. (B) The impact of circFOXK2 on the proliferation of NCI‐H1299 cells was evaluated using the EdU assay, scale bar = 50 μm. (C) Transwell assays were performed on NCI‐H1299 cells to examine the migration and invasion abilities after knockdown or overexpression of circFOXK2. *p<0.05; **p<0.01; ***p<0.001. [file CAM4-14-e70729-s003.tif]

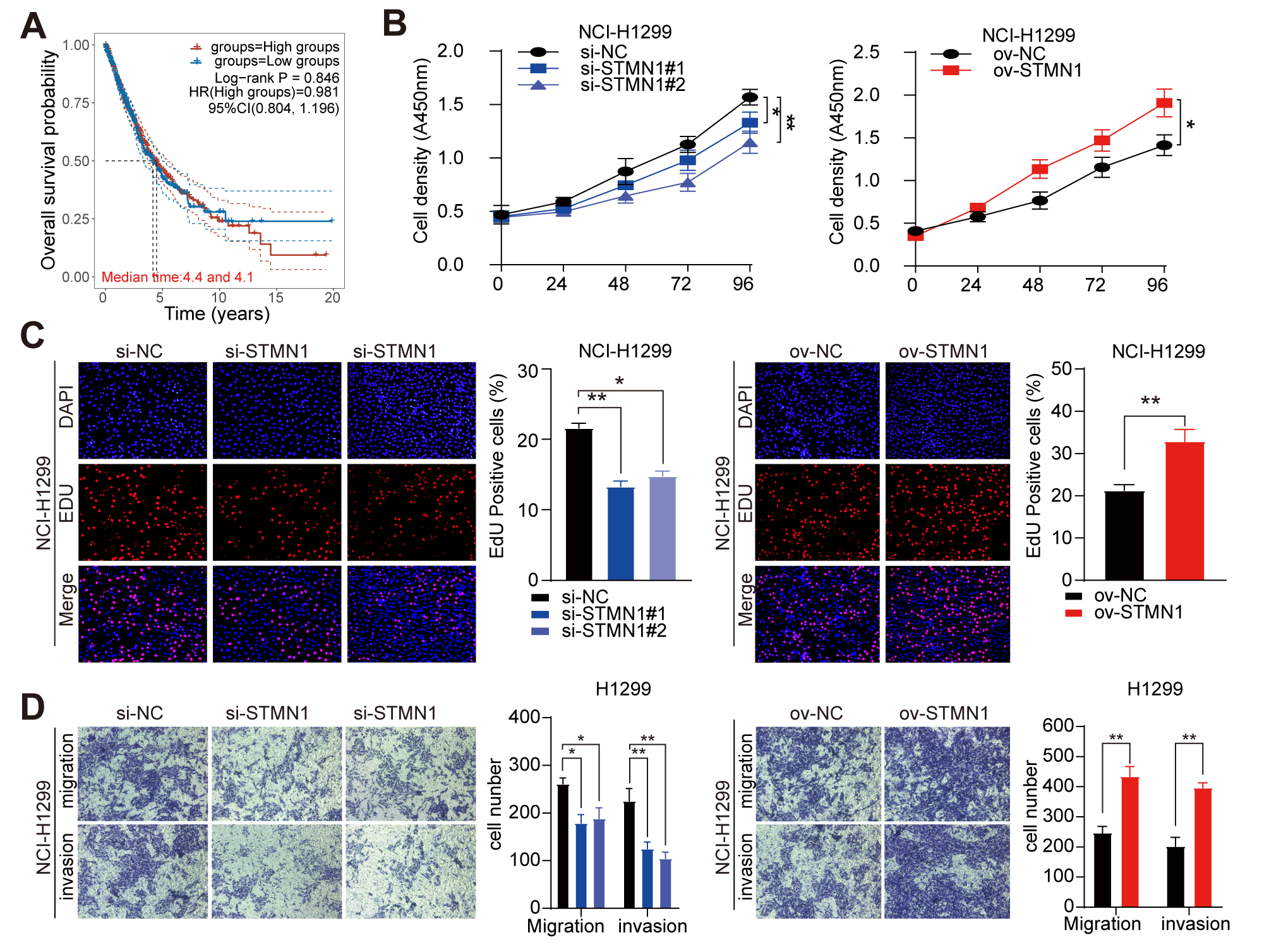

Supplement: Supplementary file 2 — Figure S2. STMN1 promotes NSCLC cells proliferation, migration, and invasion in vitro. (A) Survival analysis of NSCLC patients based on STMN1 expression in the TCGA database. (B) The growth curve of NCI‐H1299 cells was evaluated using the CCK‐8 assay after STMN1 knockdown or overexpression. (C) The impact of STMN1 on the proliferation of NCI‐H1299 cells was evaluated using the EdU assay, with a scale bar of 50 μm. (D) The migration and invasion capabilities of NCI‐H1299 cells after STMN1 knockdown or overexpression were examined by Transwell assay. *p<0.05; **p<0.01; ***p<0.001. [file CAM4-14-e70729-s004.tif]

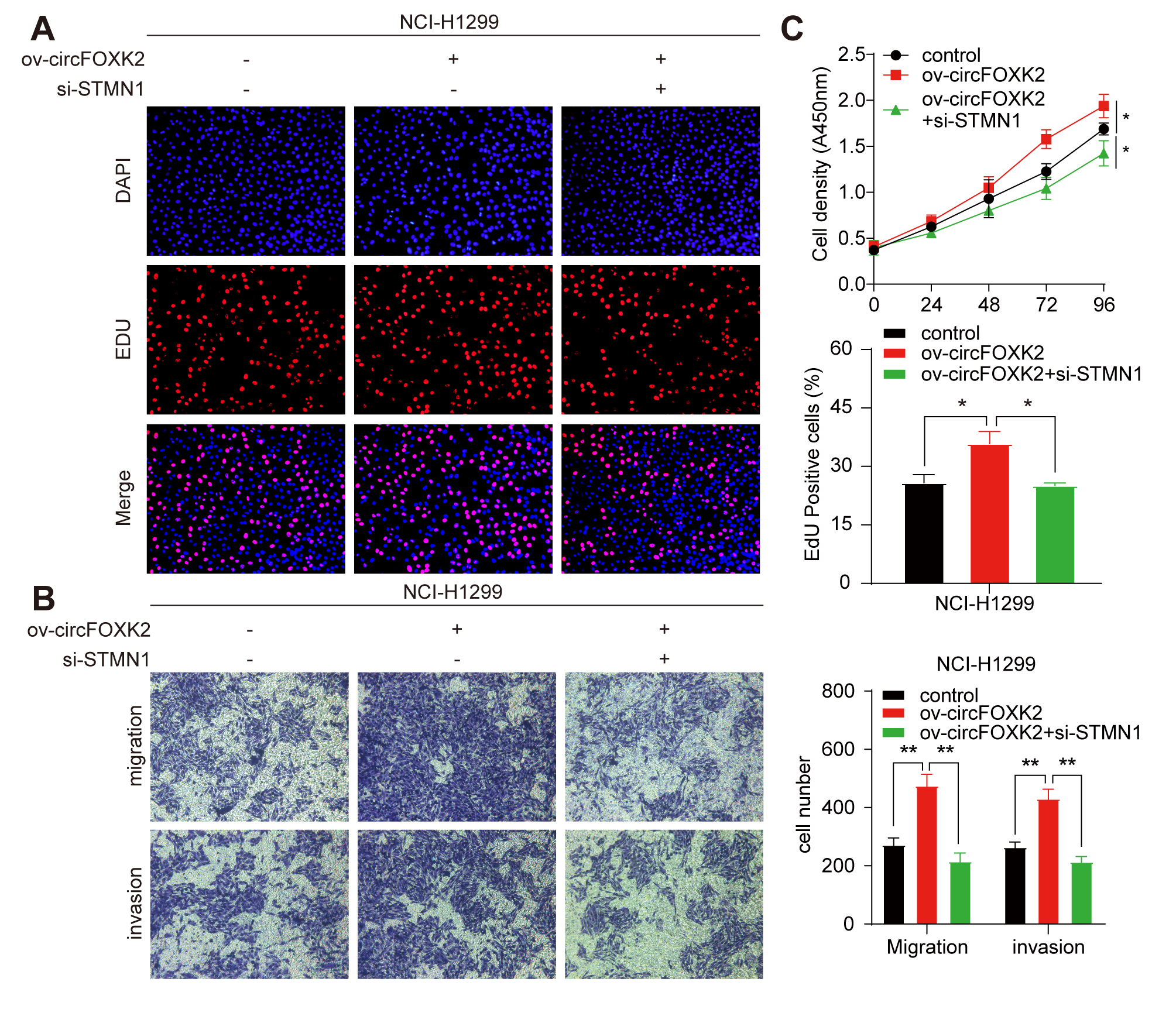

Supplement: Supplementary file 3 — Figure S3. CircFOXK2 promotes the proliferation and metastasis of NSCLC by regulating STMN1. (A, C) EdU assays and CCK‐8 experiments indicated that co‐transfection with circFOXK2 overexpression plasmid and si‐STMN1 in NCI‐H1299 cells could offset the promotional effect caused by the overexpression of circFOXK2. (B) Transwell assays of NCI‐H1299 cells demonstrated that STMN1 knockdown restored the enhanced effects on migration and invasion due to overexpression of circFOXK2. *p<0.05; **p<0.01; ***p<0.001. [file CAM4-14-e70729-s001.tif]

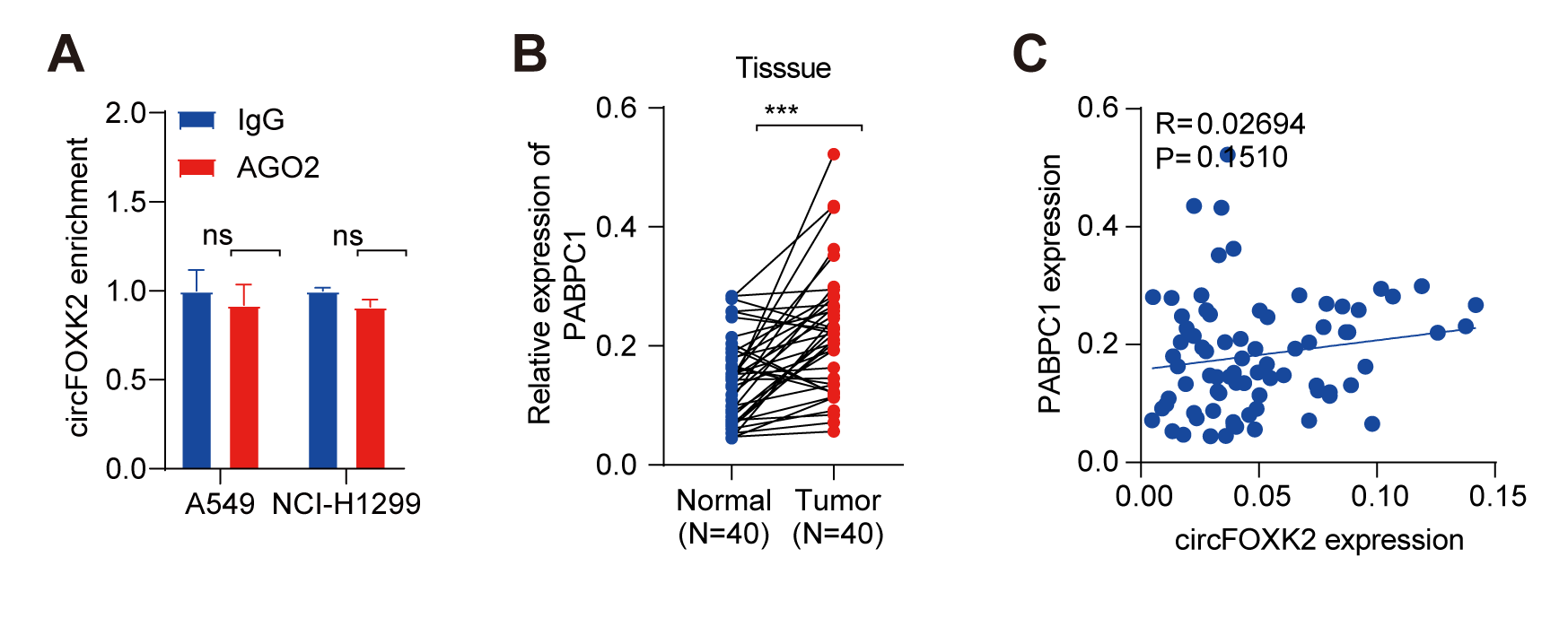

Supplement: Supplementary file 4 — Figure S4. (A) RIP assay was used to detect the interaction between AGO2 and circFOXK2 in A549 and NCI‐H1299 cells. (B) qRT‐PCR was employed to measure the relative expression of PABPC1 in 40 pairs of NSCLC tissues. (C) The correlation between circFOXK2 and PABPC1 was validated in 40 cases of NSCLC tissues. *p<0.05; **p<0.01; ***p<0.001 [file CAM4-14-e70729-s002.tif]
