# Supplementary material for: circFOXK2 Stabilizes STMN1 mRNA via PABPC1 to Promote the Progression of NSCLC
Source: Cancer Med. 2025 Feb 27;14(5):e70729. doi: 10.1002/cam4.70729 (PMC11866311; doi:10.1002/cam4.70729)
Supplement: Supplementary file 5 — Table S1. Primer sequences. Table S2. RNA oligonucleotide sequences. [file CAM4-14-e70729-s005.docx]

**Table S1** Primer sequences.

| Name | **Forward primer (5’ to 3’)** | **Reverse primer (5’ to 3’)** |
| --- | --- | --- |
| circFOXK2 sanger | AACACAAAACGGCACTCACG | TTTGTGCTCGGGAACCTGAA |
| circFOXK2 convergent | GGCACCTCTAGGTCAACACC | ATGTTTGTGCTCGGGAACCT |
| circFOXK2 divergent | TCAGGCGATTACGATGGCTC | TTGATGGCCTGTGGTAGCTG |
| FOXK2 | AAAGGAAGCGTCAGGTGGAG | GGCTAATTGCAGGGACAGGT |
| STMN1 | CAGCTAGAGAGTGCCACCAC | CTGGGCTCAGGGTATGTTCA |
| PABPC1 | CATCCTCTCCATCCGGGTC | CTGCTGGAAGTTCACATACGC |
| GAPDH convergent | GAGTCAACGGATTTGGTCGT | GACAAGCTTCCCGTTCTCAG |
| GAPDH divergent | GAAGGTGAAGGTCGAGTC | GAAGGTGAAGGTCGAGTC |

**Table S2** RNA oligonucleotide sequences.

| **RNA oligos** | **Sequences** |
| --- | --- |
| si-NC | Sense: 5’- UUCUCCGAACGUGUCACGUTT -3’  Anti-sense: 5’- ACGUGACACGUUCGGAGAATT -3’ |
| si-circFOXK2#1 | Sense: 5’- AGGUGAACAAUGGUGCACAUUTT -3’  Anti-sense: 5’- AAUGUGCACCAUUGUUCACCUTT -3’ |
| si-circFOXK2#2 | Sense: 5’- GUGAACAAUGGUGCACAUUCATT -3’  Anti-sense: 5’- UGAAUGUGCACCAUUGUUCACTT -3’ |
| si-circFOXK2#3 | Sense: 5’- AACAAUGGUGCACAUUCAGGUTT -3’  Anti-sense: 5’- ACCUGAAUGUGCACCAUUGUUTT -3’ |
| si-STMN1#1 | Sense: 5’- GGAUCUUAAUCAUCAACAAAUTT -3’  Anti-sense: 5’- UUGUUGAUGAUUAAGAUCCUUTT -3’ |
| si-STMN1#2 | Sense: 5’- CGAGUUCAAGAGAUCGAAACG TT -3’  Anti-sense: 5’- UUUCGAUCUCUUGAACUCGUGTT -3’ |
| si-PABPC1#1 | Sense: 5’- GGUUAGCUAGAGAAGAGAAGUTT -3’  Anti-sense: 5’- UUCUCUUCUCUAGCUAACCUATT -3’ |
| si-PABPC1#2 | Sense: 5’- GGAUUUGUCUUUAGAUCAAUUTT -3’  Anti-sense: 5’- UUGAUCUAAAGACAAAUCCUATT -3’ |
